# Tobacco consumption patterns among Iranian adults: a national and sub-national update from the STEPS survey 2021

**DOI:** 10.1038/s41598-023-37299-3

**Published:** 2023-06-24

**Authors:** Mohsen Abbasi-Kangevari, Ali Ghanbari, Nima Fattahi, Mohammad-Reza Malekpour, Masoud Masinaei, Naser Ahmadi, Seyyed-Hadi Ghamari, Mohammadreza Naderian, Mohammad-Mahdi Rashidi, Negar Rezaei, Erfan Ghasemi, Yosef Farzi, Moein Yoosefi, Nazila Rezaei, Elmira Foroutan Mehr, Mana Moghimi, Maryam Nasserinejad, Ali Maleki, Zeinab Abbasi-Kangevari, Farshad Farzadfar

**Affiliations:** 1 Non-Communicable Diseases Research Center, Endocrinology and Metabolism Population Sciences Institute, Tehran University of Medical Sciences, Tehran, Iran; 2 Department of Internal Medicine, Yale School of Medicine, New Haven, CT USA; 3 Department of Epidemiology and Biostatistics, School of Public Health, Tehran University of Medical Sciences, Tehran, Iran; 4 Tehran Heart Center, Cardiovascular Diseases Research Institute, Tehran University of Medical Sciences, Tehran, Iran; 5 Endocrinology and Metabolism Research Center, Endocrinology and Metabolism Clinical Sciences Institute, Tehran University of Medical Sciences, Tehran, Iran

**Keywords:** Disease prevention, Health policy, Public health

## Abstract

Smoking is recognised as a critical public health priority due to its enormous health and economic consequences. Constant monitoring of the effectiveness of tobacco control programs calls for timely population-based data. This study reports the national and sub-national patterns in tobacco consumption among Iranian adults based on the results from the STEPwise approach to chronic disease risk factor surveillance (STEPS) survey 2021. This study was performed through an analysis of the results of the STEPS survey 2021 which had been conducted as a nationally representative cross-sectional study. Participants included Iranian adults aged ≥ 18 years in all provinces of Iran, who were selected via multistage cluster sampling method. Data were analyzed via survey analysis while considering population weights. The total number of participants was 27,874, including 15,395 (55.23%) women and 12,479 (44.77%) men. The all-ages prevalence of current tobacco smoking was 14.01% overall, 4.44% among women, and 25.88% among men. The all-ages prevalence of current cigarette smoking was 9.33% overall, 0.77% among women, and 19.95% among men. The all-ages prevalence of current hookah smoking was 4.5% overall, 3.64% among women, and 5.56% among men. The mean (SD) number of cigarettes smoked per day was 12.41 (10.27) overall, 7.65 (8.09) among women, and 12.64 (10.31) among men. The mean (SD) monthly times of hookah use was 0.42 (7.87) overall, 2.86 (23.46) among women, and 0.3 (6.2) among men. The national all-ages prevalence of second-hand smoking at home was 24.64% overall, 27.38% among women, and 20.26% among men. The national all-ages prevalence of second-hand smoking at work was 19.49% overall, 17.33% among women, and 22.94% among men. The tobacco consumption in Iran remains alarmingly high, indicating the current tobacco control policy implementation level is ineffective and insufficient. This calls for adopting, implementing, and enforcing comprehensive packages of evidence-based tobacco control policies.

## Introduction

Smoking, the second leading risk factor for deaths and disability-adjusted life-years (DALYs), has caused more than 200 million deaths over the last three decades^1^. It has been recognised as a critical public health priority due to its enormous health and economic consequences^2^. As a modifiable risk factor, the burden attributable to smoking could be minimised via the effective implementation of tobacco control interventions. Since the introduction of the World Health Organization (WHO) Framework Convention on Tobacco Control (FCTC)^3^, countries have made endeavors to implement demand-reduction tools, including reducing affordability through taxation, passing smoke-free laws, mandating health warnings on packaging, and banning tobacco advertising, promotion, and sponsorship^4^.

Nevertheless, implementation and progress in tobacco control programs have varied substantially across countries^5^. As the success of tobacco control programs has slowed in many countries in the past decade, it is estimated that the total number of smokers continues to increase globally^1^. In this sense, constant monitoring of the effectiveness of previous tobacco control programs calls for timely population-based data on the prevalence of tobacco use for taking prompt public health measures^6^. The STEPwise approach to chronic disease risk factor surveillance (STEPS), developed by WHO, focuses on obtaining data on established risk factors that determine the burden of significant non-communicable diseases. The STEPS study has been conducted seven times in Iran in 2004, 2007, 2008, 2009, 2011, 2016 and 2021. This study reports the national and sub-national smoking prevalence and tobacco consumption among Iranian adults based on the results from the STEPS survey 2021.

## Methods

### Ethics approval

The study methodology conformed to Helsinki Declaration standards as revised in 1989. All experimental protocols were approved by the National Institute for Health Research under reference number IR.TUMS.NIHR.REC.1398.006. All participants provided written informed consent prior to participation in the study. Moreover, the data used in the study did not include any identifiable personal information of participants, and the confidentiality of the data and the results are preserved.

### Data source

Data were derived from the STEPS study 2021, a national cross-sectional survey carried out by the Non-Communicable Diseases Research Center (NCDRC). The STEPS survey required a nationally-representative sample. Five components were considered when estimating the sample size, including the confidence interval, margin of error, design effect, baseline index level, and non-response rate. The sample size for evaluating risk factors of NCDs in each province was calculated using a proportion to population size method. The survey used a systematic cluster classification method with 3176 clusters, each with ten participants, to conduct the study on the national level. Iranian adults aged above 18 residing in urban or rural areas of one of the 31 provinces were studied as the target population, excluding certain individuals such as those with mental disorders and pregnant women. In total, 28,821 participants from 3176 clusters were selected from rural and urban areas of the 31 provinces of Iran. A detailed description of the study population and the sampling method of the 2021 version of the STEPS survey has been published elsewhere^7^.

### Variables

Smoking was assessed using the transcultural-adaption of the STEPS questionnaire, as proposed by the World Health Organization (WHO) in national STEPS surveys. Covariates included sociodemographic status of participants and their underlying conditions. Outcome variables included smoking-related variables. Sociodemographic variables included age, sex, province of residence, residential area, years of schooling, marital status, employment status, health insurance, complementary insurance, and wealth. Underlying conditions included body mass index (BMI), hypertension, diabetes, and cardiovascular diseases. Smoking-related variables included forms of smoking including cigarette smoking, hookah use, pipe smoking, and smokeless tobacco use; amount of cigarette smoking; frequency of smoking; tobacco consumption; history of ex-smoking; attempt to quit smoking; history of second-hand smoking; and place of second-hand smoking. Current tobacco smoking was defined as the use of smoked tobacco products, including cigarettes, pipes, or hookah, daily, non-daily or occasional basis in the past 12 months. Second-hand smoking, or passive smoking, was defined as being exposed to the smoke of any tobacco products in the past 30 days.

### Statistical analysis

#### Data weighting

Data weighting was an important step between data cleaning and analysis in this survey. It was necessary to deal with incomplete data and non-response, as well as differences in the population characteristics. To ensure the reliability and validity of the results, a weighting procedure was used to adjust the survey data based on several factors. The four stages of the weighting procedure included: (a) weighting for general non-response, (b) weighting for non-response at each step of the survey, (c) weighting for age, sex, and area of residence, and (d) final weighting for the analysis of data. More information on the weighting process and equations used has been published elsewhere^7^.

#### Descriptive measures

Frequencies, proportions, means, and standard deviations were used to describe the data. We used the chi-square test for categorised variables. T-test and one-way analysis of variance (ANOVA) test were used to analyse the differences among means of two groups and three groups or more, respectively. We considered p-values below 0.05 as significant.

#### Age standardisation

The National Population and Housing Census conducted by Iran's Statistical Center in 2016 was considered the standard population for direct age-standardisation to allow comparisons between provinces^8^.

#### Inequity analysis

Principal Component Analysis (PCA) was applied to derive the household wealth index based on questions on key dwelling characteristics and household ownership, as described in the study protocol. PCA is an approach to statistical analysis in which multiple datasets are combined as orthogonal components^9^. The wealth index was used to divide the population into quintiles, whereby the first and fifth quintiles present the least fortunate and wealthiest households, respectively.

The concentration index with Erreygers' correction^10^ was used to quantify the degree of inequality in all types of smoking-related to wealth index and years of schooling. A zero concentration index would indicate no inequality related to wealth index or years of schooling. Negative values of concentration index would mean a higher prevalence of smoking among people with lower wealth index or years of schooling. Positive values of concentration index would indicate a higher prevalence of the type of smoking among people with higher wealth index or years of schooling^11^.

Model-based clustering method was applied to the data to achieve homogenous clusters regarding tobacco use on the sub-national level based on the smoking behaviours reported. We used data mining methods to determine the number of clusters and the combination of provinces in each cluster^12,13^.

#### Tools

All essential data analyses were performed using R statistical package v3.4.3 (http://www.r-project.org, RRID: SCR_001905). Data visualisations were performed using Python programming language, version 3.6 and R statistical package v3.4.3.

## Results

### Sociodemographic status

The total number of participants was 27,874, including 15,395 (55.23%) women and 12,479 (44.77%) men. The sociodemographic status of participants is presented in Table 1.

**Table 1:** Socio-demographic status of participants.

Variables	Women	Men	Total	p-value
Age, mean (SD)	45.29 (15.43)	46.19 (16.48)	45.69 (15.91)	< 0.001
Residential area, n (%)				
Urban	11,199 (75.15)	9077 (74.79)	20,276 (74.99)	
Rural	4196 (24.85)	3402 (25.21)	7598 (25.01)	0.523
Years of schooling, n (%)				
Zero	2825 (17.27)	1190 (8.77)	4015 (13.48)	
1–6	4048 (59.29)	2695 (40.71)	6743 (23.78)	
7–11	2490 (48.57)	2698 (51.43)	5188 (19.08)	
12+	5918 (51.39)	5804 (48.61)	11,722 (43.65)	< 0.001
Marital status, n (%)				
Divorced	414 (2.91)	152 (1.3)	566 (2.19)	< 0.001
Married	11,445 (74.25)	9914 (79.5)	21,359 (76.59)	
Single	1961 (12.49)	2286 (18.21)	4247 (15.04)	
Widow	1575 (10.35)	127 (0.98)	1702 (6.17)	
Employment status				
Freelance job or self-employed	631 (4.26)	5705 (46.94)	6336 (23.31)	< 0.001
Private sector employee	358 (2.82)	407 (3.7)	765 (3.22)	
Private Sector labor	144 (0.96)	934 (7.27)	1078 (3.77)	
Public sector employee	583 (3.81)	1070 (8.22)	1653 (5.78)	
Public sector labor	44 (0.29)	210 (1.61)	254 (0.88)	
Retired	381 (2.65)	2157 (17.58)	2538 (9.31)	
Unemployed due to disability	48 (0.28)	398 (2.83)	446 (1.42)	
Unemployed Not seeking work	117 (0.77)	201 (1.69)	318 (1.18)	
Unemployed seeker job	222 (1.42)	562 (4.22)	784 (2.67)	
Unpaid work	12,753 (82.75)	743 (5.94)	13,496 (48.46)	
Basic health insurance, n (%)				
Yes	14,003 (91.05)	10,966 (87.87)	24,969 (89.63)	
No	1278 (8.95)	1421 (12.13)	2699 (10.37)	< 0.001
Complementary insurance, n (%)				
Yes	4262 (28.97)	3346 (27.76)	7608 (28.42)	
No	10,960 (71.03)	8975 (72.24)	19,935 (71.58)	0.05
BMI				
< 18.5	500 (3.2)	469 (3.58)	969 (3.37)	< 0.001
18.5–24.9	4451 (28.76)	4974 (39.62)	9425 (33.61)	
25–29.9	5641 (36.83)	4844 (39.59)	10,485 (38.06)	
30–34.9	3286 (21.63)	1702 (13.93)	4988 (18.19)	
35–39	1086 (7.2)	338 (2.68)	1424 (5.18)	
40+	352 (2.4)	70 (0.6)	422 (1.6)	
Hypertension				
Yes	5135 (32.93)	3896 (30.92)	9031 (32.03)	0.001
No	10,207 (67.07)	8510 (69.08)	18,717 (67.97)	
Diabetes				
Yes	1456 (14.71)	987 (13.45)	2443 (14.15)	
No	8829 (85.29)	6832 (86.55)	15,661 (85.85)	0.095
Cardiovascular diseases				
Yes	941 (6.11)	1105 (9.05)	2046 (7.42)	
No	14,428 (93.89)	11,348 (90.95)	25,776 (92.58)	< 0.001

### Prevalence of tobacco use

#### Prevalence of tobacco use among women and men of all ages

The all-ages prevalence (95% CI) of current tobacco smoking was 4.44% (4.09–4.82) among women, 25.88% (25.03–26.75) among men, and 14.01% (13.56–14.48) overall. The all-ages prevalence of current cigarette smoking was 0.77% (0.62–0.95) among women, 19.95% (19.17–20.75) among men, and 9.33% (8.95–9.72) overall. The all-ages prevalence of current hookah smoking was 3.64% (3.33, 3.98) among women, 5.56% (5.12–6.03) among men, and 4.5% (4.23–4.78) overall. The all-ages prevalence of all types of tobacco smoking among both sexes is presented in Table 2.

**Table 2:** All-age prevalence of all types of tobacco smoking among both sexes.

Smoking type		Women		Men		Total		p-value
		%	95% CI	%	95% CI	%	95% CI	
Ever smoking	Ever tobacco smoking	7.23	6.79–7.7	34.59	33.66–35.53	19.44	18.93–19.97	< 0.001
	Ever cigarette smoking	1.21	1.02–1.43	26.06	25.21–26.93	12.31	11.88–12.75	< 0.001
	Ever use of hookah	6.2	5.8–6.64	10.51	9.92–11.13	8.13	7.78–8.49	< 0.001
	Ever pipe smoking	0.03	0.01–0.09	0.6	0.46–0.78	0.28	0.22–0.37	< 0.001
	Ever use of smokeless tobacco	0.1	0.06–0.16	1.17	0.98–1.39	0.57	0.49–0.68	< 0.001
Current smoking	Current tobacco smoking	4.44	4.09–4.82	25.88	25.03–26.75	14.01	13.56–14.48	< 0.001
	Current cigarette smoking	0.77	0.62–0.95	19.95	19.17–20.75	9.33	8.95–9.72	< 0.001
	Current use of hookah	3.64	3.33–3.98	5.56	5.12–6.03	4.5	4.23–4.78	< 0.001
	Current pipe smoking	0.01	0–0.07	0.16	0.09–0.26	0.08	0.05–0.12	< 0.001
	Current use of smokeless tobacco	0.08	0.04–0.13	0.82	0.67–1.01	0.41	0.34–0.5	< 0.001
Ex-smoking	Ex-cigarette smoking	0.45	0.34–0.58	6.11	5.66–6.6	2.98	2.76–3.21	< 0.001
	Ex use of hookah	2.56	2.29–2.86	4.95	4.54–5.4	3.63	3.39–3.89	< 0.001
	Ex pipe smoking	0.02	0.01–0.07	0.44	0.33–0.6	0.21	0.16–0.28	< 0.001
	Ex use of smokeless tobacco	0.02	0.01–0.07	0.34	0.25–0.47	0.17	0.12–0.22	< 0.001
Second-hand smoking	Second-hand smoking at home	27.38	26.59–28.18	20.26	19.39–21.17	24.64	24.05–25.24	< 0.001
	Second-hand smoking at work	17.33	16.67–18.02	22.94	22.01–23.9	19.49	18.95–20.05	< 0.001
	Second-hand smoking at home and work	14.3	13.69–14.93	11.55	10.86–12.27	13.24	12.78–13.71	< 0.001

*Not significant.

#### Age pattern of tobacco use prevalence

**Figure 1:**
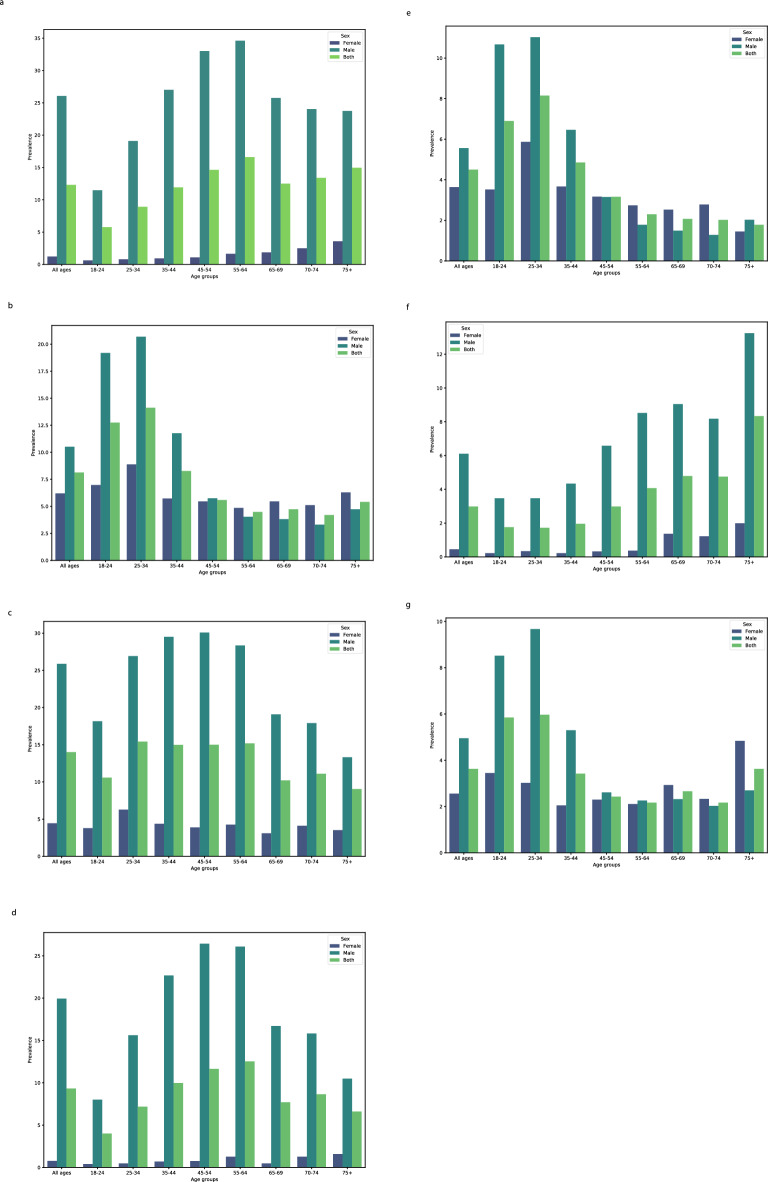
The prevalence of tobacco use among various age group: ever cigarette smoking (**a**); ever use of hookah (**b**); current smoking (**c**); current cigarette smoking (**d**); current use of hookah (**e**); ex-cigarette smoking (**f**); ex use of hookah (**g**).

The prevalence (95% CI) of current cigarette smoking among men increased with age, from 8.01% (6.45–9.91) among men aged 18–24 to the observed peak of 26.43% (24.47–28.48) among age-group 45–54 years; however, it then decreased to 10.49% (8.08–13.51) among those aged more than 75. The prevalence of current use of hookah reached the observed peak of 11.03% (9.66, 12.56) among men and 5.87% (4.98, 6.9) women aged 25–34, then decreased to 2.03% (1.1–3.72) among men aged more than 75 and 2.53% (1.66–3.85) among women aged 65–69. Some 19.19% (16.83–21.78) and 6.97% (5.63–8.62) of men and women aged 18–24 years had used hookah at least once. The corresponding rates were 20.7% (18.91–22.61) and 8.88% (7.77–10.13) for men and women aged 25–34 years, respectively (Fig. 1, Supplementary Table 1).

#### Geographical pattern of tobacco prevalence

**Figure 2:**
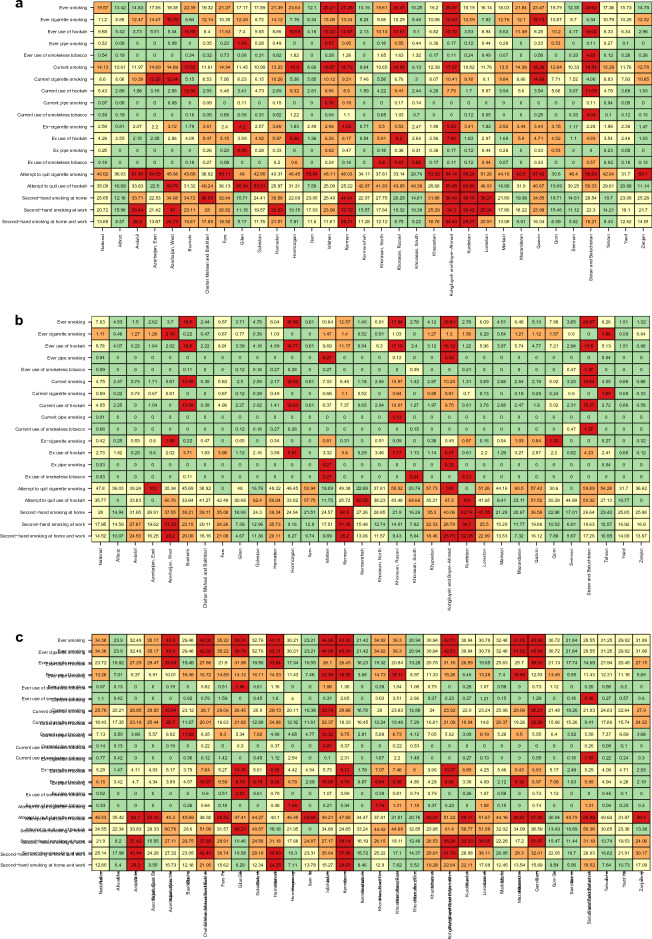
The geographical pattern of tobacco use prevalence in Iran: total (**a**); women (**b**); men (**c**).

The age-standardised (95% CI) prevalence of all types of smoking varied significantly across the 31 provinces of Iran (Fig. 2a–c, Supplementary Table 2).

#### Geographical pattern of tobacco prevalence among women

The age-standardised prevalence (95% CI) of ever smoking among women ranged from 0.81% (0.19–3.35) in Ilam to 20.87% (17.62–24.54) in Sistan and Baluchistan. The prevalence of ever cigarette smoking among women ranged from Zero percent in Ilam, Hormozgan, Sistan and Baluchistan, and South Khorasan to 2.19% (1.19–4) in West Azerbaijan. The prevalence of ever use of hookah among women ranged from 0.23% (0.03–1.59) in Ardabil to 19.5% (16.38–23.06) in Sistan and Baluchistan. The prevalence of current smoking among women ranged from 0.39% (0.12–1.29) in Chahar Mahaal and Bakhtiari to 16.64% (13.69–20.08) in Sistan and Baluchistan. The prevalence of current cigarette smoking among women ranged from zero percent in Bushehr, Chahar Mahaal and Bakhtiari, Hormozgan, Ilam, North Khorasan Sistan, South Khorasan, Markazi, and Baluchistan to 1.59% (1.04–2.43) in Tehran. The prevalence of the current use of hookah among women ranged from zero percent in Ardabil and West Azerbaijan to 15.27% (12.47–18.56) in Sistan and Baluchistan (Fig. 2b, Supplementary Table 2).

#### Geographical pattern of tobacco prevalence among men

The age-standardised prevalence (95% CI) of ever smoking among men ranged from 20.94% (16.19–26.65) in South Khorasan to 45.99% (39.71–52.39) in Qazvin. The prevalence of ever cigarette smoking among men ranged from 13.28% (9.65–18.01) in South Khorasan to 39.02% (33.05–45.34) in Qazvin. The prevalence of ever use of hookah among men ranged from 3.68% (1.96–6.81) in Kermanshah to 22.38% (12.63–20.63) in Isfahan. The prevalence of current smoking among men ranged from 12.88% (9.31–17.57) in South Khorasan to 38.21% (32.25–44.55) in Qazvin. The prevalence of current cigarette smoking among men ranged from 7.29% (4.77–10.97) in South Khorasan to 32.39% (26.87–38.44) in Qazvin. The prevalence of current use of hookah among men ranged from 2.81% (1.38–5.66) in Kermanshah to 12.32% (9.31–16.12) in Isfahan (Fig. 2c, Supplementary Table 2).

### Cigarette and hookah smoking

#### Cigarette and hookah smoking among women and men

**Figure 3:**
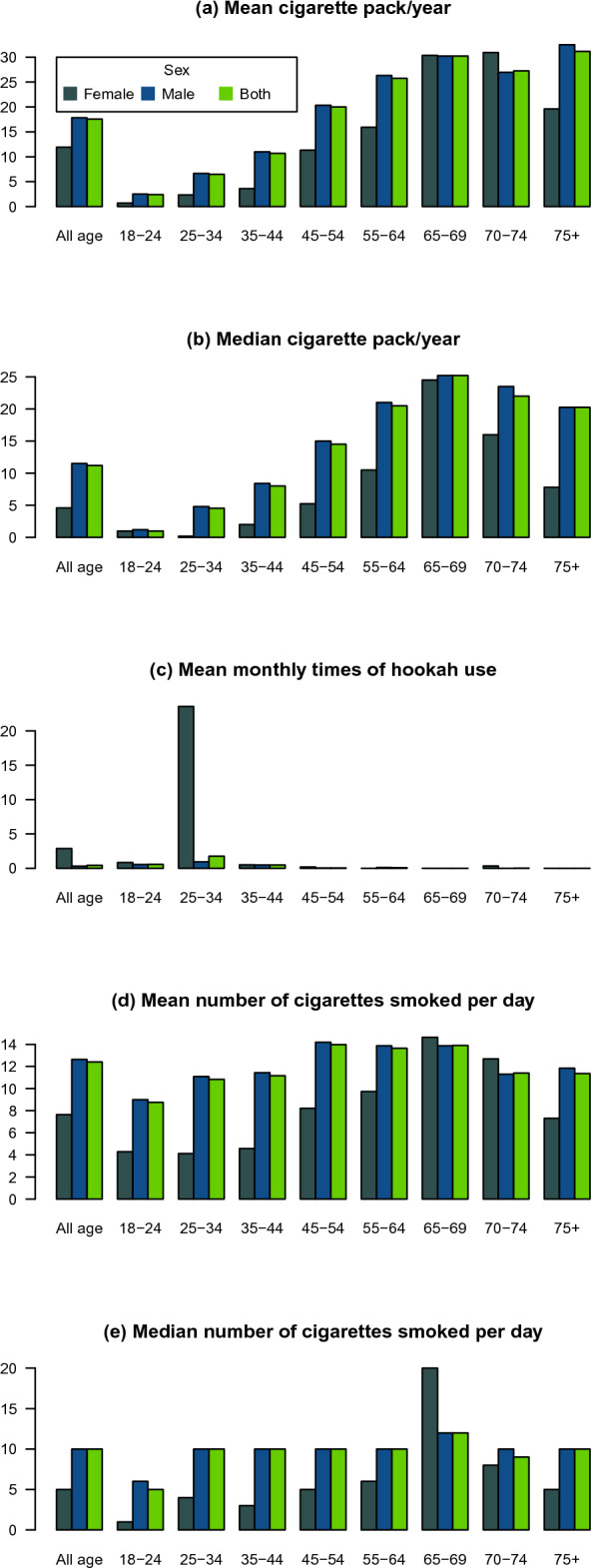
Tobacco consumption among various age groups: mean cigarette pack/year (**a**); median cigarette pack/year (**b**); mean monthly times of hookah use (**c**); mean number of cigarettes smoked per day (**d**); median number of cigarettes smoked per day (**e**).

The all-ages mean (SD) cigarette pack/year was 17.56 (19.32) overall, 11.93 (17.35) among women, and 17.83 (19.37) among men. The mean (SD) number of cigarettes smoked per day was 12.41 (10.27) overall, 7.65 (8.09) among women, and 12.64 (10.31) among men. The mean (SD) monthly times of hookah use was 0.42 (7.87) overall, 2.86 (23.46) among women, and 0.3 (6.2) among men (Fig. 3, Supplementary Table 3).

#### Age pattern of cigarette and hookah smoking

The mean (SD) number of cigarettes smoked per day increased with age, from 9 (9) among men and 4.28 (5.02) among women aged 18–24 to the observed peak of 13.87 (10.08) among men and 14.64 (7.73) among women aged 65–69 (Fig. 3, Supplementary Table 3).

#### Geographical pattern of cigarette and hookah smoking

**Figure 4:**
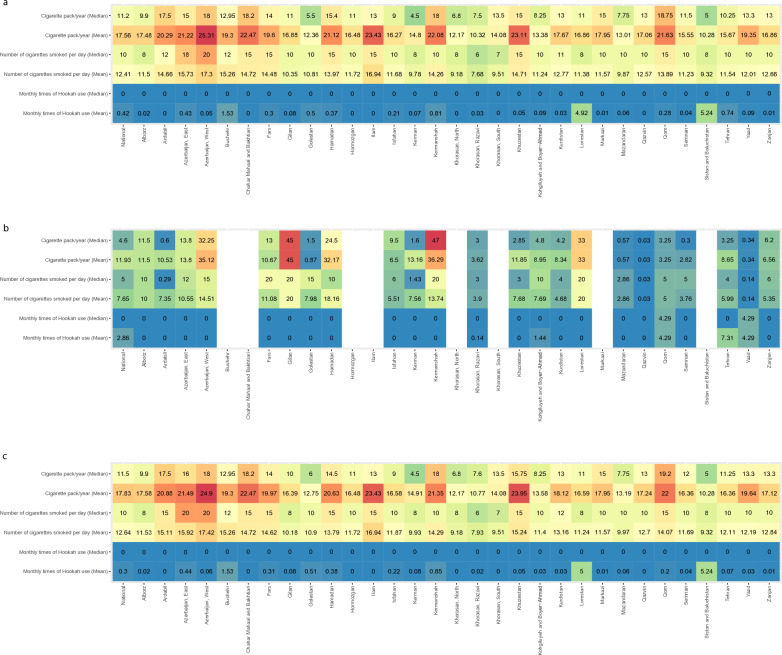
The geographical pattern of tobacco consumption in Iran: total (**a**); women (**b**); men (**c**).

Cigarette and hookah smoking varied significantly among men and women across the 31 provinces of Iran (Fig. 4a–c, and Supplementary Table 4).

#### Geographical pattern of cigarette and hookah smoking among women

Among provinces with enough data, the mean cigarette pack/year ranged from 0.03 in Qazvin to 45 in Gilan. The median cigarette pack/year ranged from 0.03 in Qazvin to 45 in Gilan. The mean number of cigarettes smoked per day ranged from 0.03 in Qazvin to 18. 61 in Hamadan. The median number of cigarettes smoked per day ranged from 0.03 in Qazvin to 20 in Kermanshah, Gilan, Lorestan, and Fars. The mean monthly times of hookah use ranged from Zero in Alborz, Ardebil, East Azerbaijan, West Azerbaijan, Fars, Gilan, Golestan, Hamadan, Isfahan, Kerman, Kermanshah, Khuzestan, Lorestan, Mazandaran, Qazvin, Semnan, and Zanjan to 7.31 in Terhan. The median monthly times of hookah use ranged from Zero in Alborz, Ardebil, East Azerbaijan, West Azerbaijan, Fars, Gilan, Golestan, Hamadan, Isfahan, Kerman, Kermanshah, Razavi Khorasan, Khuzestan, Kurdistan, Kohgiluyeh and Boyer − Ahmad, Lorestan, Mazandaran, Qazvin, Semnan, Tehran and Zanjan to 4.29 in Qom and Yazd (Fig. 4b, Supplementary Table 4).

#### Geographical pattern of cigarette and hookah smoking among men

The mean cigarette pack/year ranged from 10.28 in Sistan and Baluchistan to 24.9 in West Azerbaijan. The median cigarette pack/year ranged from 4.5 in Kerman to 19.2 in Qom. The mean number of cigarettes smoked per day ranged from 7.93 in Razavi Khorasan to 17.4 in West Azerbaijan. The median number of cigarettes smoked per day ranged from 6 in Razavi Khorasan to 20 in West Azerbaijan and East Azerbaijan. The mean monthly times of hookah use ranged from zero in Ardebil, Chahar Mahaal and Bakhtiari, Hormozgan, Ilam, North Khorasan, South Khorasan, and Qazvin to 5.24 in Sistan and Baluchistan. The median monthly times of hookah use was zero in all provinces (Fig. 4a, Supplementary Table 4).

### Prevalence of second-hand smoking

#### Prevalence of second-hand smoking at home

**Figure 5:**
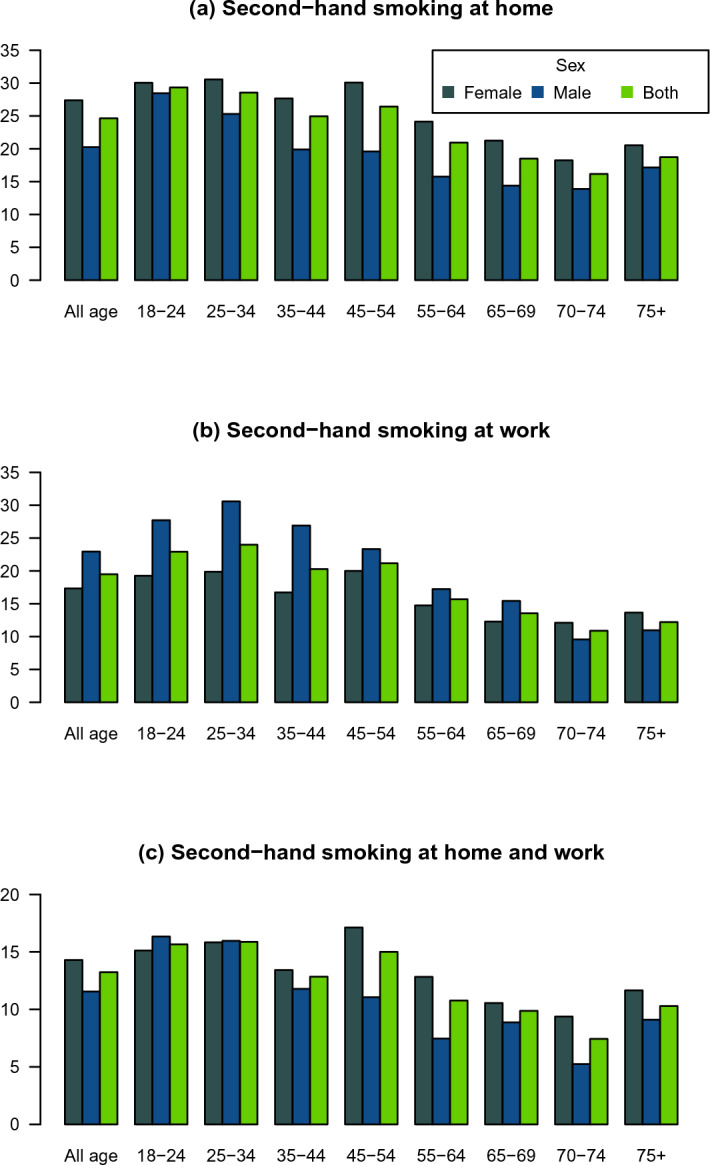
Prevalence of second smoking among various age groups: at home (**a**); at work (**b**); at home and work (**c**).

The national all-ages prevalence (95% CI) of second-hand smoking at home was 24.64 (24.05–25.24) overall, 27.38 (26.59–28.18) among women, and 20.26 (19.39–21.17) among men. The prevalence of second-hand smoking at home decreased with age among both sexes, from 29.35% (27.39–31.39) among age-group 18–24 to 16.17% (13.74–18.93) among those aged 70–74. It then rose to 18.72% (16.22–21.52) among those aged more than 75. The prevalence of second-hand smoking at home among women ranged from 14.94% (11.11–19.79) % in Alborz to 48.9% (43.5–54.33) in Kerman. The prevalence of second-hand smoking at home among men ranged from 8.2% (5–13.17) in Alborz to 39.14% (32.84–45.83) in Kerman (Figs. 2, 5, Supplementary Tables 1, 2).

#### Prevalence of second-hand smoking at work

The national all-ages prevalence (95% CI) of second-hand smoking at work was 19.49% (18.95–20.05) overall, 17.33% (16.67–18.02) among women, and 22.94% (22.01–23.9) among men. The prevalence of second-hand smoking at work among both sexes increased from 22.92% (21.11–24.84) among age-group 18–24 to 23.98% (22.62–25.39) among age-group 25–34. The prevalence of second-hand smoking at work among women ranged from 6.91% (4.47–10.53) in Semnan to 36.7% (31.91–41.77) in Kurdistan. The prevalence of second-hand smoking at work among men ranged from 14.37% (10.06–20.1) in South Khorasan to 43.44% (35.72–51.49) in Ardabil (Figs. 1, 5, Supplementary Tables 1, 2).

### Determinants of tobacco smoking

#### Wealth and years of schooling

**Figure 6:**
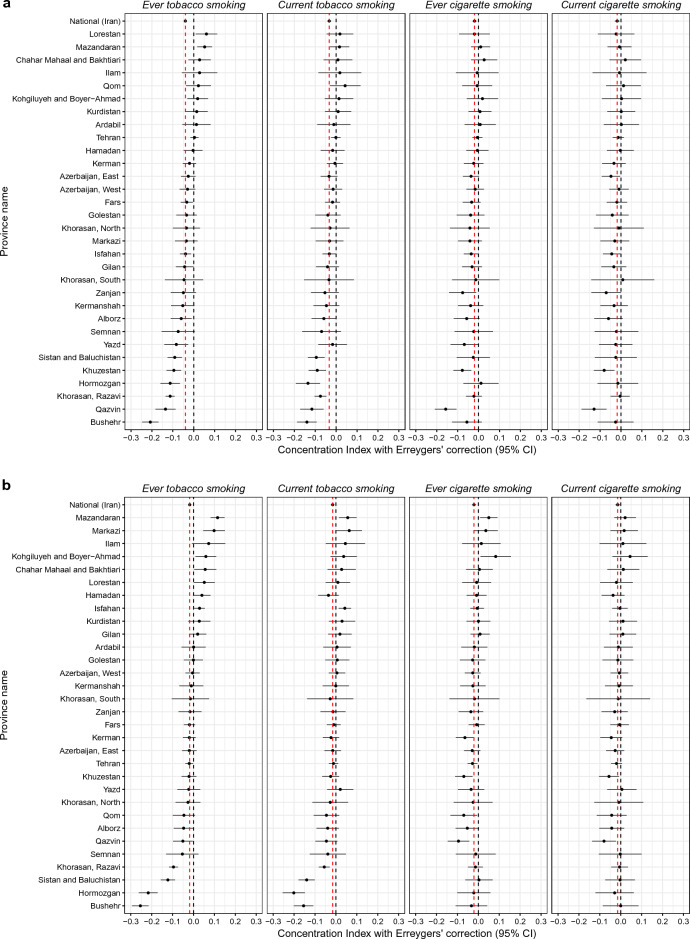
National and subnational concentration index for wealth (**a**) and years of schooling (**b**) for tobacco and cigarette smoking among women and men.

On the national level, lower wealth index or years of schooling were associated with higher prevalence of ever or current tobacco smoking among both women and men (Table 3). Nevertheless, wealth index and years of schooling had varying roles in tobacco smoking prevalence among women and men across provinces in Iran (Fig. 6, Supplementary Table 5).

**Table 3:** Concentration index for wealth and years of schooling for tobacco and cigarette smoking among women and men.

Type of smoking	Concentration index	
	Wealth index	Years of schooling
Ever tobacco smoking		
Female	−0.043 (−0.06, −0.025)	−0.028 (−0.045, −0.011)
Male	−0.069 (−0.076, −0.061)	−0.093 (−0.101, −0.086)
Total	−0.04 (−0.047, −0.033)	−0.018 (−0.025, −0.011)
Ever cigarette smoking		
Female	−0.004 (−0.051, 0.043)	−0.007 (−0.054, 0.04)
Male	−0.066 (−0.075, −0.058)	−0.12 (−0.128, −0.111)
Total	−0.019 (−0.029, −0.01)	−0.021 (−0.03, −0.012)
Current tobacco smoking		
Female	−0.03 (−0.052, −0.008)	−0.016 (−0.038, 0.005)
Male	−0.062 (−0.071, −0.053)	−0.084 (−0.093, −0.075)
Total	−0.033 (−0.042, −0.024)	−0.016 (−0.024, −0.007)
Current cigarette smoking		
Female	−0.001 (−0.063, 0.061)	−0.002 (−0.065, 0.062)
Male	−0.06 (−0.071, −0.05)	−0.095 (−0.105, −0.084)
Total	−0.018 (−0.029, −0.007)	−0.015 (−0.025, −0.004)

#### Geographical pattern

**Figure 7:**
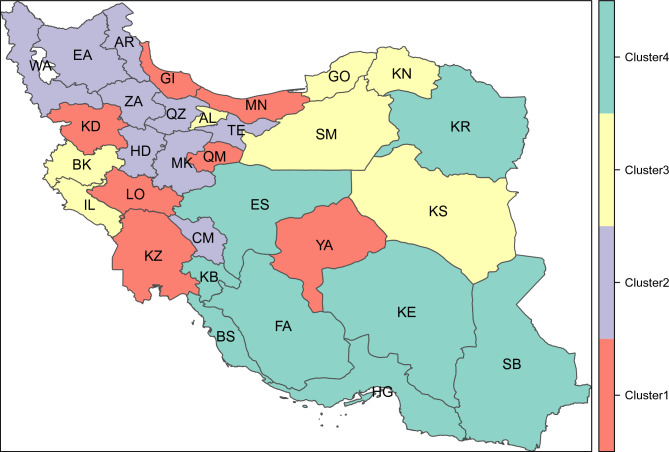
Model based clustering of Iran 31 provinces into four clusters based on prevalence of ever tobacco smoking, ever cigarette smoking, current tobacco smoking, and current cigarette smoking. *AL* Alborz, *AR* Ardebil, *EA* Azerbaijan-East, *WA* Azerbaijan-West, *BS* Bushehr, *CM* Chahar-Mahal-Bakhtiari, *FA* Fars, *GI* Gilan, *GO* Golestan, *HD* Hamedan, *HG* Hormozgan, *IL* Ilam, *ES* Isfahan, *KE* Kerman, *BK* Kermanshah, *KN* North Khorasan, *KR* Razavi Khorasan, *KS* South Khorasan, *KZ* Khuzestan, *KB* Kohkiluyeh-and-Boyer-Ahmad, *KD* Kurdistan, *LO* Lorestan, *MK* Markazi, *MN* Mazandaran, *QZ* Qazvin, *QM* Qom, *SM* Semnan, *SB* Sistan-and-Baluchestan, *TE* Tehran, *YA* Yazd, *ZA* Zanjan.

Using model-based clustering, the 31 provinces of Iran were categorized into four clusters based on prevalence of ever tobacco smoking, ever cigarette smoking, current tobacco smoking, and current cigarette smoking (Fig. 7). The prevalence of smoking among the four clusters is presented in Table 4.

**Table 4:** The prevalence of smoking among the four clusters of Iran 31 provinces.

Type of smoking	Cluster 1	Cluster 2	Cluster 3	Cluster 4
Ever tobacco smoking	17.19	18.69	13.71	24.91
Ever cigarette smoking	12.32	15.01	9.17	11.07
Current tobacco smoking	11.81	14.19	9.71	17.23
Current cigarette smoking	8.61	11.97	6.89	7.86

## Discussion

The study showed that 14% of the Iranian adults, 4.4% of women and 25.9% of men, were current tobacco smokers in 2021. Some 9.3% of the study participants, 0.8% of women and 20% of men, were current cigarette smokers. Moreover, the all-ages prevalence of current hookah use was 3.6% among women and 5.6% among men. Pipe smoking and smokeless tobacco were unpopular among the Iranian population.

The prevalence of current tobacco smoking since the previous STEPS survey in 2016 has stagnated: form 14.1% in 2016 to 14% in 2021. Nevertheless, the mean number of cigarettes smoked per day has increased from 10.4 in 2016 to 12.4 in 2021^14^.

There was a heterogeneous geographical distribution pattern in tobacco smoking prevalence in Iran, distinctively witnessed among the smoking behaviour of women and men, depending on the type of smoking. In northwestern Iran, West Azerbaijan had the second-highest prevalence of current cigarette smoking among men and the fourth-highest among women. Nevertheless, it ranked 16th in terms of current hookah use among men, and the prevalence was nearly zero among women. In southwestern Iran, Bushehr had the highest prevalence of current hookah use among both women and men, while having nearly zero prevalence of current cigarette smoking among women and being ranked as the third-lowest in terms of prevalence among men. In southeastern Iran, women in Sistan and Baluchistan had the second-highest prevalence of current hookah use while having nearly zero prevalence of current cigarette smoking. Sistan and Baluchistan also had the highest prevalence of smokeless tobacco use. The geographical pattern also conformed to neighbouring countries^1,15^, possibly due to ethnic, cultural, and access resemblance^16^.

Using data mining techniques, we grouped Iran’s provinces into four clusters with similar prevalence of ever tobacco smoking, ever cigarette smoking, current tobacco smoking, and current cigarette smoking. In provinces in cluster 1, smoking cessation policies need to be prioritized, while other forms of tobacco use, namely hookah are less pressing issues. In cluster 2, which interestingly maps to northwestern Iran, both cigarette smoking and tobacco use need to be addressed. In provinces in cluster 3 both ever/current prevalence of tobacco and cigarette smoking are low. Cluster 4 is consistent with central, southern, and eastern provinces, where tobacco consumption needs to be addressed. The clustering of provinces could empower Iranian authorities in decision-making and public policy efforts regarding tobacco control. This needs to be further investigated in future studies which particularly focus on proposing tobacco reduction policies based on the real-world data. Notably, upon interpretation of the results of model-based clustering, the role of smoking determinants reported in this study including wealth index and years of schooling need to be considered for policy and intervention development.

The prevalence gap between men and women was narrower in the current hookah use compared with current cigarette smoking. The prevalence of hookah use is more than cigarette smoking among women. Moreover, women tend to have more positive attitudes towards hookah use, which is much less stigmatised than cigarette smoking^17^. In recent years, the prevalence of hookah use among women has increased more than men^18^. Alarmingly, the study showed that women's mean monthly times of hookah use was more than nine times that of men. Meanwhile, there is evidence that the deleterious effects of hookah use on women could be higher than in men^19^.

There was an interest in hookah use, particularly among younger adults. Some 19% and 7% of men and women aged 18–24 years had used hookah at least once. Moreover, the prevalence of current hookah use reached a surprisingly early peak of 11% among men and 6% women aged 25–34. Despite supposedly more deleterious effects of hookah use than cigarette smoking^20^, there has been a growing interest in hookah use in recent decades^21^. While hookah use is considered recreational in Iran^16^, there is evidence that hookah users are increasingly susceptible to cigarette smoking^22,23^.

Six decades after the first documentations of the deleterious health effects of tobacco use^24^, there has been substantial progress in reducing the prevalence of tobacco smoking worldwide. In contrast, it is estimated that the age-standardised prevalence of current tobacco smoking has increased by 8% among men and 2% among women in the past three decades in Iran and the population growth has also led to a marked increase in the number of smokers^1^. Since introducing the WHO Framework Convention on Tobacco Control (FCTC) in 2005, outlining demand-reduction policies, many counties have witnessed drastic decreases in the prevalence of tobacco smoking^25^. Taking a closer look at the previous STEPS surveys in Iran, the smoking prevalence of Iranian adults has somewhat stagnated since 2004 among both men and women^26^. Simultaneously, Brazil, Norway, and Senegal have managed to decrease the prevalence of tobacco smoking by 73%, 54%, and 51%, respectively. Iceland, Denmark, Canada, Australia, Colombia, and Costa Rica, also witnessed nearly 50% decreases in the prevalence of tobacco smoking^1^.

Though once an early adapter to FCTC^16^, the current tobacco control policy implementation level is insufficient in Iran. In the absence of any new concerted effort towards smoking reduction, sanctions and subsequently the economic downturn could have played a significant role in the slight reduction of smoking prevalence in Iran since 2016^14,16,27^. Evidence shows that economic recessions are associated with decreased cigarette consumption via decreasing its affordability^16^. Overall, cigarettes have become less affordable in Iran, particularly since 2018^28^. The purchase pattern of Iranian smokers has changed from the whole box of cigarettes to the single stick cigarette. In addition, smokers have generally swapped to less expensive cigarettes^29^. Dealing with the burden attributable to tobacco use, public health authorities encounter substantial obstacles such as an increased number of smokers due to population growth, pressure from the tobacco industry, and competing for health and political priorities^1^. WHO discourages Iran from implementing high import duties as they encourage domestic production of tobacco. Direct taxation also needs to be excised rather than other indirect taxes.

Moreover, uniform tax rates are recommended to avoid product switching^28^. There is evidence that tobacco taxation, as one of the most cost-effective tobacco control policies^30^, needs to be concordantly adjusted to people's purchasing power to remain potent and reduce affordability^31^. Moreover, the revenue from tobacco taxation needs to be redistributed to tobacco control programs, health care services, and social support services^30^. Unless strict regulations are in place to control cigarette smuggling, any increase in cigarette price could be compensated by flooding smuggled cigarettes into the market^29^. This calls for substantially reducing smoking rates in the country via adopting, implementing, and enforcing comprehensive packages of evidence-based tobacco control policies. The witnessed heterogeneities and determinants in the patterns of smoking prevalence and tobacco consumption need to be taken into account when prioritizing vulnerable groups and designing tobacco reduction strategies, especially considering the relative authority of the medical universities of Iran in sub-national healthcare programs implementation^32^. Otherwise, the death toll, imposed economic costs, and the burden to health systems caused by smoking will increase over the years.

### Strengths and limitations

The main strength of this study lies in its large sample size. Given the sampling method, the results could represent the Iranian population. The study investigated the smoking prevalence and tobacco consumption pattern by age, sex, province of residence, wealth, and years of schooling. This could empower public health authorities to make evidence-based decisions and design tobacco reduction action plans based on various determinants according to real-world data, especially considering that individual level data were used for assessing the inequality patterns. Moreover, this was the first nationwide study from Iran to investigate pipe smoking and the use of smokeless tobacco. Given the use of electronic and online data gathering tools, the study had very little missing data. To minimise the missing data, the software used for data collection was designed to avoid ignoring obligatory questions. Moreover, the estimated sample size was initially calculated to be 10% higher than the required sample size so that potential missing data did not violate the sample representativeness.

## Conclusion

The tobacco consumption in Iran remains alarmingly high, indicating the current tobacco control policy implementation level is insufficient. This calls for adopting, implementing, and enforcing comprehensive packages of evidence-based tobacco control policies. In the meantime, the witnessed heterogeneities in the patterns of smoking prevalence and tobacco consumption need to be considered when designing tobacco reduction strategies. Otherwise, the death toll, imposed economic costs, and the burden to health systems caused by smoking will increase over the years.

## Supplementary Information

Supplementary Table 1.

Supplementary Table 2.

Supplementary Table 3.

Supplementary Table 4.

Supplementary Table 5.

## Data Availability

The datasets used and analysed during the current study available from the corresponding author on reasonable request.
